# To Tell, or Not to Tell; Confidentiality in an Iranian HIV Positive Patient: A Viewpoint 

**Published:** 2017-03

**Authors:** Mahshad Noroozi, Maliheh Kadivar, Mansure Madani, Pooneh Salari

**Affiliations:** 1 Medical Ethics and History of Medicine Research Center , Tehran University of Medical Sciences, Tehran, Iran; Department of Medical Ethics, School of Medicine, Tehran University of Medical Sciences, Tehran, Iran; 2 Division of Neonatology, Department of Pediatrics, Children’s Medical Center, Tehran University of Medical Sciences, Tehran, Iran; 3 Medical Ethics and History of Medicine Research Center, Tehran University of Medical Sciences, Tehran, Iran

**Keywords:** Confidentiality, HIV, Ethics, Sexual Partner

## Abstract

Confidentiality is a basic concept in medical ethics and protecting confidentiality is considered as physicians’ duty. In some occasions, this protection is in conflict with the right of the patient’s sexual partner, who should be informed about the possibility of being infected. The sexual partner being pregnant, the situation is going to be more complicated. In this paper, we present a case discussion with special ethical, legal, social, cultural, and religious aspects. According to this informing sexual partner with the patient’s assent, opt-out Human immunodeficiency virus (HIV) screening in pregnant women and enhancing psychosocial and family support are highly recommended. Strategic changes in health system policies and regulations seem to be necessary as well.

## Introduction

Respecting patients’ privacy and confidentiality originates from the principle of respecting human dignity and patient’s autonomy. Privacy refers to the boundaries between an individual and others, while confidentiality manifests the limitation of access to one’s information. Privacy is the patients’ right, but confidentiality is the physicians’ duty. Confidentiality and privacy are connected to each other. In medical practice, privacy is a kind of relationship between physician and patient, and confidentiality is determined when a third party is involved in the physician-patient relationship. Confidentiality empowers trust in physician-patients relationship, and has a crucial role in stability of this relationship (1). In some situations, keeping patient’s information confidential and respecting his privacy by the physician is in conflict with benefits of the third party. For example confidentiality for the information of a Human immunodeficiency virus (HIV) positive patient may be in conflict with his partner’s benefit. In this article, we try to discuss this potential conflict with particular consideration of the Iranian culture. At the end, we will present a solution, based on our social and cultural background.

Scenario

A two week old male infant was admitted to hospital with frequent vomiting. He was hospitalized in the neonatal intensive care unit (NICU) for seizure, dehydration, and electrolytesim balances. His medical history showed that the baby was born by cesarean section from a 23 year old nulliparous mother with birth weight of 2850 grams in a hospital on Tehran suburb. He was the first child of aconsanguineous marriage. His father was an IV drug user and was passed away before baby’s birth, because of acquired immune deficiency syndrome (AIDS) and tuberculosis. After father’s death, the mother was informed about her husband’s AIDS. At this time, the mother′s HIV testing (who was in the third trimester of pregnancy) showed positive and antiretroviral treatment was started. After delivery, antiretroviral therapy was initiated for the new born and continued for his mother. Because of depression, the mother stopped taking her medications one week prior to the infant’s admission. She was living with her family without any support from her spouse’s relatives. 

During hospitalization, the newborn was comprehensively evaluated for sepsis secondary to intra-uterine infections, including Toxoplasmosis, Rubella, Cytomegalovirus (CMV) and Herpes infections (TORCH), and for other infectious diseases such a syphilis, varicella-zoster, parvovirus B19, along with HIV. Broad-spectrum antibiotics and intravenous phenobarbital was started for him. According to the family history, infectious consultation suggested treatment with zidovudine. Finally, he was discharged onco-trimoxazole and zidovudine on 27^th^ day of his life. 

The mother was referred to the social worker service and HIV follow up center for psychological and further evaluation and support.

Ethical and legal considerations:

According to this scenario, both the pregnant mother and the fetus are considered to be victims of keeping an HIV positive patient′s confidentiality. In this case, in addition to the mother’s best interests, baby’s health and well-being should be considered. If the mother was aware of her husband’s AIDS, her decision about pregnancy might have been different. Even if this HIV positive woman wanted to fall pregnant, she would have needed special assessments and the viral load should have been decreased as much as possible (2). The most important ethical concept in this case was patient’s confidentiality which was considered unconditional. When the father being alive, was the breaching confidentiality ethically permissible based on his wife’s interests and her health concerns? Should we keep confidentiality after father’s death? Does the responsibility of health care professionals extend to the patient’s family, including his partner and his child? What are the consequences of breaching patient’s confidentiality to his partner?

There are complex ethical issues which should be considered in regards to an HIV positive patient confidentiality. Most HIV treatment strategies allow patients to act arbitrarily about the diagnostic tests, treatment and consultation. Therefore, breaching confidentiality may cause patients’ fear for referring to the health care system; which obviously delays diagnosis and treatment (1, 2). If patients believe that their secrets will be kept confidential by the physician, they share their private information with their physician such as high-risk behaviors and addiction. This trust has to be maintained in all circumstances (3). 

The argument is not limited to the patient and we must also consider the third parties’ interests, if the patient is married or has a sexual partner. Hence, there is another issue: If the third parties health is related to his/her partner, is still patient’s confidentiality considered to be unconditional? To answer this question, we present two approaches;

As the first approach, after diagnosis, the patient enters in a support system. The patient is consulted and notified for the probability of virus transmission to her/his partner. The patient will be encouraged to inform the partner otherwise he/she will give assent to the health system to inform the partner. After notifying, the third party will undergo the HIV testing. In the absence of infection, she will take necessary preventive measures, otherwise antiretroviral treatment will be started for him/her. In this approach, if the patient refuses to notify his/her partner for any reason, the partner will not be informed. In some countries including Iran, France and Thailand this approach is preferred.

The second approach emphasizes on the third party’s notification as an ethical duty (4). Based on the recommendation of Center for Disease Control (CDC), after diagnosis, sexual partner should be notified within 2-3 working days. Many states of America have enacted rules, which legally recommend patients and physicians to inform the third party about the risk of infection (5). In this approach, third party interest is one of the most important points that should be considered in confidentiality, therefore breaching confidentiality has been allowed in some cases (3). Nevertheless, it is highly recommended to inform the third party by the patients assent. This approach is historically based on Tarasoff case in the 1970s, which led to the formation of Tarasoff law in the United States of America. In this situation, it is physician’s duty to consider third parties safety and to give warning (6). Some countries like Ukraine, Australia and United States act in this way.

This case presents a situation in which the third party’s interest affected patient’s confidentiality. Thus, medical associations and organizations such as the American Medical Association (AMA) has changed their predominant traditional viewpoint about confidentiality, and their statement expresses “*when a patient threatens to inflict serious physical harm to another person and there is a reasonable probability that the patient may carry out the threat, the physician should take reasonable precautions for the protection of the intended victim” *(7). Can we apply this argument to HIV partner notification? It seems that physician’s ethical duty necessitates notifying the third party when there is a high risk of infection and the patient do not accept to inform his partner (8). In this case, the husband had high risk behaviors (intravenous drug use) accompanied with AIDS manifestations. Hence, AMA’s definition of serious physical injury is become applicable (7). 

Breaching confidentiality in contagious diseases not only benefits the third party but also is in line with public benefits. Public interest is one of the reasons cited in the literature as a condition of breaching confidentiality. Early diagnosis and prevention can increase patient’s survival and decrease health costs. Unidentified third party will increase the number of infected patients over time and more financial resources will be consumed. The British Medical Association (BMA) also considered the transmission of serious infectious disease to the community as a convincing factor in breaching confidentiality (9). 

Accordingly, we can ethically justify HIV partner notification based on public viewpoints and third party interests. According to the article 648 of the Penal Code of Iran, disclosure of patients’ secrets by medical team is an offense, which can be associated with legal consequences and punishments. Therefore, physicians’ fear of legal consequences is a major limitation in informing HIV-positive patients’ spouse. 

Psycho-socio-religious considerations

In addition to the problems caused by the disease, HIV infection has psycho-social burdens. According to the White House Office of National AIDS Policy, stigma associated with the disease and its related behaviors is considered as one of the major barriers to prevent HIV epidemic (10). Breaching confidentiality in HIV/AIDS is not completely accepted because of social stigma, and discrimination (4). Scambler et al. presented stigma as multidimensional phenomena which blemishes social personality (11). 

High risk sexual behaviors are considered as one way of HIV transmission and are morally and religiously unacceptable in many cultures such as Iranian. HIV-positive patients may be stigmatized for these types of behaviors which create social fear and major obstacle in notifying their partners (12). Considering the socio-religious context of Iran, stigmatization and discrimination of HIV-positive patients is one of the major obstacles for disclosure (4). 

Furthermore, patients’ network and family support may provide proper psychosocial support. In Iranian society, individuals are highly bounded to their family, and their social status is not separated from their families which may originate from traditional and religious believes. In this context the Iranians do not welcome inappropriate behaviors (from both moral and religious point of view) and may profoundly stigmatize HIV-positive patients. 

High risk and illegal sexual behaviors are prohibited in Islam. It seems that HIV is less prevalent in Muslim countries due to the Islamic teachings. However because of potential stigmatization, HIV-positive patients tend to not disclose their health conditions even with their partner to prevent dishonor (4). To avoid this rejection and the discrimination, some HIV-positive patients stop treatment which put them at the increased risk of HIV transmission. Therefore, in this context the role of the social support system becomes more highlighted. 

Recent statistical information shows that until 2014, there were only 29000 registered HIV-positive cases in Iran and drug usage is among the most common way of transmission. As the result of the fear for stigmatization and dishonor it is estimated that there are more than 80000 unregistered HIV positive cases in Iran, which is a potential source of transmission. Of 29000 HIV positive patients in 2013, one third were women and unprotected sexual relationship was the way of transmission in 36% of HIV-positive women. It is suggested that in Iran, sexually transmitted HIV infections are on the rise which will change the pattern of transmission (13). 

Medical considerations

The other important aspect in HIV diagnosis, prevention and treatment is HIV screening during pregnancy. 

***Vertical transmission of HIV from mother to child:*** Placenta is a major obstacle in HIV transmission from mother to baby. Studies show that HIV transmission does not occur in the first trimester of pregnancy. Because of micro-transfusion in maternal-fetal blood, HIV transmission mostly happens in the third trimester especially few weeks before birth. Maximum transmission of virus takes place in labor through mucosal contact with infected mother’s blood or secretions. Cesarean section before labor pain reduces the risk of transmission up to 50% (14). 

Based on some studies, the risk of virus transmission by breast milk is very low (0.6%-0.8%). World Health Organization (WHO) recommends starting triple regimen at 14^th^ weeks of gestational age. If three medications are not available all together, Zidovudine can be used to prevent disease transmission. In order to reduce virus transmission to fetus, treatment is continued with a single dose of Novirapin at the beginning of childbirth. Zidovudine in combination with lamivudine is prescribed during childbirth and should be continued for 7 days. A single dose of Novirapin is prescribed for the baby initially and for 6 to 12 hours after birth. Zidovudine prophylaxis is prescribed and continued for 4-6 weeks (15). Based on clinical conditions of mother and availability of medications, the World Medical Association (WHO) provided other therapeutic protocols.

***HIV screening in pregnant women:*** Elimination of HIV and increasing access to treatment for infected children are WHO objectives. Therefore, every HIV-positive born baby represents a defect in prenatal care. An important goal for the health care system is to reduce HIV transmission from mother to child, which is possible by identifying and treating pregnant women (16). HIV diagnosis before or during pregnancy is a prerequisite for this purpose. HIV screening for pregnant women is still a challenge especially in Iran. Statistics published in 2013, showed that 54% of women in poor and developing countries have no access to HIV testing (17). From 33 of the states in America, 31% of mothers had no HIV testing during pregnancy (17). As a result of the change in approaches by various countries, statistics for HIV testing in pregnant women have increased from 26% in 2009 to 44% in 2013 (17). 

There are two approaches for HIV screening in pregnant women (18, 19):

1. Opt-in approach or obtaining informed consent; the HIV testing is performed after consultation with pregnant women. After her informed consent, blood samples are taken.
2. Opt-out approach or presumed consent; in this approach HIV testing is a routine screening test in pregnancy. It is assumed that pregnant women do not oppose to the HIV testing. If they do not want to be tested, they have to declare their opposition.

In the United States, studies showed that use of Opt-out approach has increased HIV testing and had positive impact on detection and prevention of mother-child HIV infection. In 2006, CDC presented suggestion about HIV testing in Morbidity and Mortality Weekly Report, and recommended opt-out approach in pregnant women. The Second HIV testing was also recommended in third trimester, preferably before 36 weeks, in high-risk mothers such as drug addicts, and those with multi sexual partners. Rapid testing during labor was advised to be done for any woman with undocumented HIV status (20). Because of the importance and the aftereffect on future life, when pregnant women refuses testing, reasons should be explored and necessary consultation and support should be provided (19, 20). In many developed and developing countries and even in some African countries, where HIV infection is a major problem, opt-out approach has been accepted especially for pregnant women and has increased HIV diagnosis (21). According to WHO, these strategies have decreased vertical HIV transmission from 26% in 2009 to 17% in 2012 (16).

The Iranian guideline on prevention of mother to child HIV transmission has been derived from WHO and the CDC's recommendations. Although the need for HIV screening in pregnant women is emphasized, it does not clearly address the opt-out screening approach (22). Accordingly, the guideline recommends that every expecting mother to be screened in the first prenatal visit. In addition the guideline emphasizes repeated screening in the third trimester especially in at risk mothers. Recently, in some provinces of Iran the opt-out HIV screening program has been performing as pilot (22). Moreover because of the potential concerns about vertical HIV transmission, some gynecologists perform HIV screening in pregnant women regardless of their consent, which is obviously unethical.

## Conclusion

This case presents the complete confidentiality for an IV drug user who is infected with HIV and his pregnant spouse was consequently infected. Due to the lack of HIV screening in Iran, and many other obstacles which were discussed in this article, the baby was born with HIV. This case presents failure in identifying positive HIV in a pregnant woman before third trimester, as well as in providing the appropriate prenatal care. There are several potential reasons for these failures including father’s fear of discrimination and stigmatization especially because of socio-religious aspects, lack of proper social support for the vulnerable in Iranian society, prohibition of breaching patients’ confidentiality based on ethical, legal and religious considerations, and lack of public awareness.

It seems that some strategic changes and modifications are necessary. First of all, changes in health system policies on HIV detection and prevention can prevent similar cases. HIV partner notification needs special attention from our health and legal systems. Secondly, this field requires multidimensional support system such as health, moral, psychology, religious, social, and family support, which are essential to overcome the ethical and legal issues. Regardless of supportive infrastructure, leading new approaches has a lot of consequences, such as patients mistrust and not attending for diagnosis and treatment of their disease. Therefore, health care system should revise the old approach which cannot solve all problems by itself. Increasing public awareness about the communicable infectious diseases especially those with the potency of vertical transmission is one of our recommendations. 

Public teaching on the disease itself and its way of transmission or on health policies can help disease prevention and avoid high-risk behaviors. Furthermore, the cultural believes based on Islamic teachings should be modified to prevent stigmatization and discrimination.
